# Superior Stability of Hydroxysafflor Yellow A in Xuebijing Injection and the Associated Mechanism

**DOI:** 10.3390/molecules22122129

**Published:** 2017-12-02

**Authors:** Weiling Pu, Huijie Zhang, Meng Wang, Yanan Liu, Lili Sun, Xiaoliang Ren

**Affiliations:** 1 Key Laboratory of Pharmacology of Traditional Chinese Medicine Formulae, Ministry of Education, Tianjin University of Traditional Chinese Medicine, Tianjin 300193, China; Linlin8229@163.com (W.P.); mengwangr@163.com (M.W.); 2 College of Traditional Chinese Medicine, Tianjin University of Traditional Chinese Medicine, Tianjin 300193, China; jyhuijie@163.com (H.Z.); tianyinan2007@163.com (Y.L.); 18322050489@126.com (L.S.)

**Keywords:** Hydroxysafflor yellow A, Xuebijing injection, degradation kinetics, stability, stabilizing effect

## Abstract

Hydroxysafflor yellow A (HSYA) is the main bioactive ingredient of XBJ injection. At first, the stability of HSYA in solution and in a Xuebijing injection was investigated, then the mechanisms of the increased stability of HSYA in the XBJ injection were investigated to provide useful information on clinical safety. HSYA stability was investigated as a function of pH and temperature in aqueous solution and an XBJ injection, following the guidelines from the International Conference on Harmonisation of Technical Requirements for Registration of Pharmaceuticals for Human Use. Products were identified by UPLC-MS/MS. HSYA reaction followed first-order kinetics under all conditions. The half-life of HSYA in XBJ was almost 40 times longer than in aqueous solution. The activation energies of HSYA reaction in aqueous solution and XBJ were calculated to be 78.53 and 92.90 kJ∙mol^−1^ by using Arrhenius equation. The results indicated that HSYA was more stable in XBJ than in aqueous solution. Two products were identified and the mechanism was intra-molecular nucleophilic substitution. The excellent stability of HSYA in XBJ injection partly due to the micelles formed in the injection. The study may provide clues for compatibility in TCM prescription and also provide useful information for further preparation technology research of HSYA and assessment of clinical safety of XBJ.

## 1. Introduction

Hydroxysafflor yellow A (HSYA) is a pharmacologically active chalcone found in *Flos Carthami*, a medicinal and edible herb from Eurasia. HSYA exhibits a variety of pharmacological properties, which are mostly mediated by its anti-inflammatory activity [1,2,3]. HSYA suppresses oxygen glucose deprivation (OGD)-induced inflammatory responses in BV2 microglia, likely by inhibiting the NF-κB signaling pathway and p38 phosphorylation [4]. Xuebijing (XBJ) injection is an intravenous Traditional Chinese Medicine (TCM) injection containing *Flosc Carthami* and four other herbs including *Radix Paeoniaec Rubra, Radix Salviae Miltiorrhizae, Radix Angelicae Sinensis*, and *Rhizoma Chuanxiong*. It demonstrates therapeutic effects on sepsis, acute pancreatitis and pulmonary infection [5,6,7] and is the first herbal TCM product used to treat sepsis [8]. HSYA is the main bioactive ingredient and a quality control marker of XBJ injection [9,10,11].

**Figure 1:**
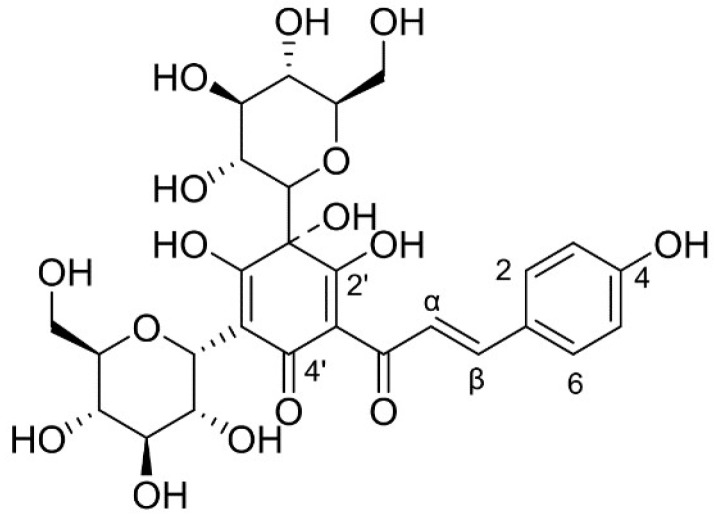
Chemical structure of HSYA.

Stability of active ingredients of herbal drugs is one of important factors affecting the quality, safety and efficacy of herbal drugs [12]. Degradation kinetics is also a common method for evaluating the stability of active ingredients under stress conditions [13,14]. Being a chalcone (Figure 1), HSYA may have poor thermo and pH stability and may therefore degrade [15,16,17,18]. To the best of our knowledge, there are few reports on the chemical stability of HSYA in aqueous solution or in XBJ. As an important TCM injection, the therapeutic efficacy and safety of XBJ have caused extensive concern. We sought to investigate the stability of HSYA in aqueous solution and in XBJ injection, which will help us to know more about the influence of preparation technology of XBJ on the stability of HSYA and will provide useful information related to the clinical safety of XBJ.

In this work, the effects of temperature and pH on HSYA stability were investigated following the guidelines of the International Conference on Harmonization of Technical Requirements for Registration of Pharmaceuticals for Human Use (ICH) [19]. The parameters associated with the degradation kinetics of HSYA in aqueous solution and XBJ under above condition were estimated. To investigate the intermolecular interactions which affect the stability of HSYA in XBJ, the XBJ injection was separated on an SPE column into water-soluble XBJ (WSX) and excipient excluded XBJ (EEX) fractions. The effects of WSX and EEX and four bioactive ingredients of XBJ (paeoniflorin (PF), danshensu (DSS), ferulic acid (FA) and senkyunolide I (Sen I)) on the degradation rate of HSYA were also determined. Finally, HSYA degradation pathways and the factors underlying its influenced stability in XBJ were proposed accordingly.

## 2. Results and Discussion

### 2.1. Method Validation

According to the calibration curve, the linear range of HSYA detection was 2–102 μg·mL^−1^ and a calibration equation of Y = 24183X + 7361 (r = 0.9998, *n* = 7) was obtained. The precision (RSD at 51 μg·mL^−1^) and the average recovery were calculated to be 0.8% and 100.53 ± 1.8% (*n* = 6), respectively.

### 2.2. HSYA Degradation Follows First-Order Reaction Kinetics

**Figure 2:**
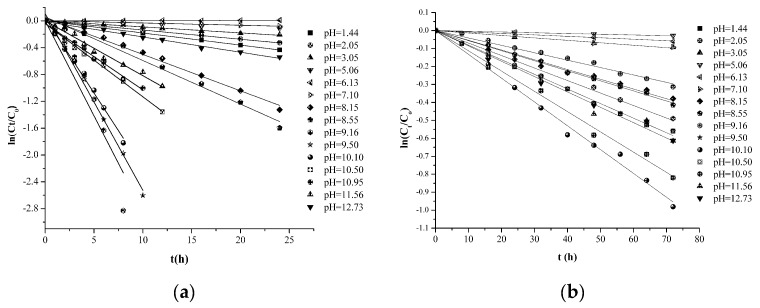
First-order plots for HSYA degradation at 25 °C in aqueous solution (**a**) and XBJ injection (**b**).

At a given initial concentration and temperature, the changes in HSYA concentration versus incubation time at different pHs are shown in Figure 2. The linear relationship between ln(C_t_/C_0_) and incubation time indicated that HSYA degradation followed the first-order reaction kinetics. The *k* was calculated using equation [20,21]:

ln (C_t_/C_0_) = *k*t (1)

where C_t_, C_0_, t and *k* represent the HSYA concentration (μg·mL^−1^) detected at incubation time t, the initial HSYA concentration (μg·mL^−1^), incubation time (h) and the observed rate constant (h^−1^), respectively. The high correlation coefficients (r^2^ > 0.97 in most cases) of the lines fitted to the experimental data indicated that the degradation of HSYA in both aqueous solution and XBJ followed the first-order kinetics quite well.

### 2.3. The pH Profile of HSYA Stability

**Figure 3:**
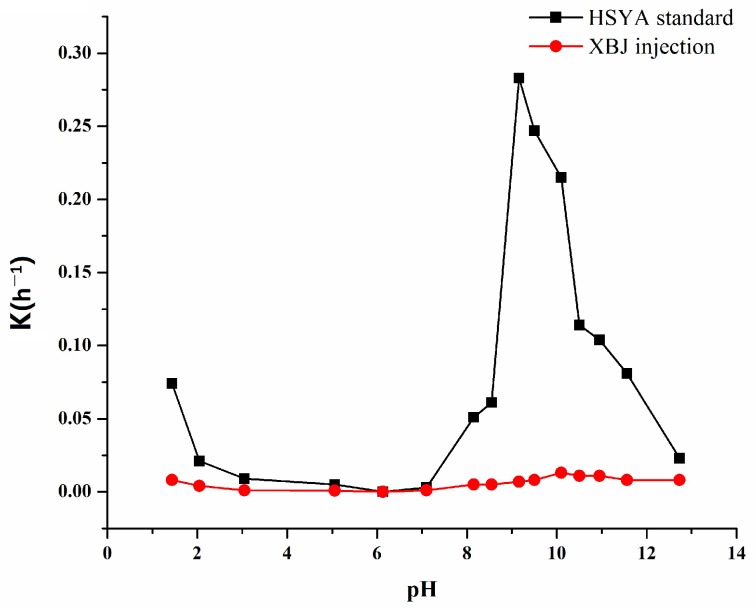
pH profile of *k* of HSYA degradation at 25 °C in aqueous solution and XBJ injection.

The pH profile of HSYA stability (*k*) followed an inverted V-shaped curve as shown in Figure 3. The *k* linear regression coefficients and t_0.5_ of HSYA at different pH in aqueous solution and XBJ injection were given in Table 1. The degradation rate increased with increasing pH under acidic conditions (pH < 6.13) and decreased with increasing pH under alkaline conditions (pH 8–9). Interestingly, under strong alkaline conditions (pH > 9), the *k* again increased with increasing pH. HSYA was most unstable at pH9 in aqueous solution. Importantly, HSYA was much more stable in XBJ injection than in aqueous solution under all conditions tested, especially under alkaline conditions.

### 2.4. The Temperature Profile of HSYA Stability

**Figure 4:**
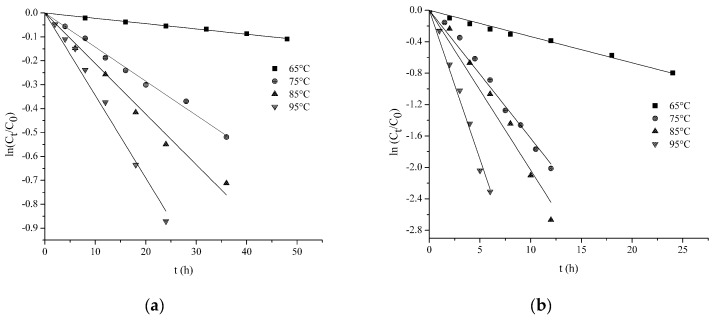
First-order plots for HSYA degradation in aqueous solution (**a**) and XBJ injection (**b**).

The changes in HSYA concentration versus time at different temperatures (65–95 °C) are shown in Figure 4. The *k* values of HSYA degradation in aqueous solution and XBJ at pH 7.10 are presented in Table 2. High correlation coefficients of linear regression analysis indicated that in both aqueous solution and XBJ, HSYA degradation under all temperatures tested followed first-order reaction kinetics. Also, HSYA degradation rate (*k*) increased with increasing temperature in both aqueous solution and XBJ and HSYA was much more stable in XBJ than in aqueous solution at any given temperature.

The activation energy of HSYA degradation was calculated using the Arrhenius equation (Equation (2)):

(2) $$\ln k_{obs}=\ln A-Ea/RT$$

where *A* represents the frequency factor, *R* (8.314 J·K^−1^·mol^−1^) is the universal gas constant, *Ea* (J·mol^−1^) is the activation energy and *T* is the absolute temperature (K). The activation energies of HSYA degradation in aqueous solution and XBJ were calculated to be 78.53 and 92.90 kJ∙mol^−1^, respectively. Higher activation energy indicates that a compound needs to acquire a greater amount of energy to undergo a specific reaction [22]. The higher *Ea* in XBJ suggested that HSYA degradation is more sensitive to temperature changes in XBJ.

### 2.5. Mass Balance

**Figure 5:**
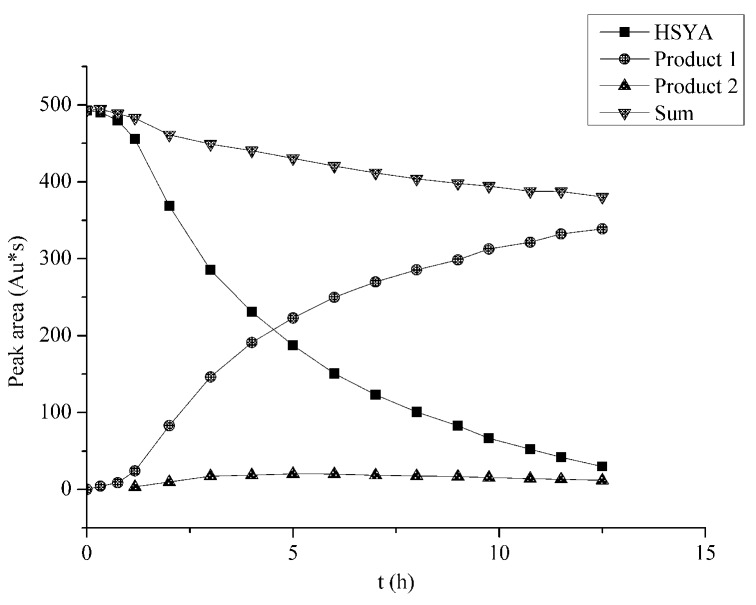
Mass balance of HYSA and the two degradation products at pH 9.16.

In the reaction, mass balance was explored. In our studies, mass balance was calculated using the peak areas:

(3) $$\mathrm{Mass}\;\mathrm{balance}\;=\;\frac{\sum^{\text{​}}Area_{i,x}}{\sum^{\text{​}}Area_{R,0}}\times 100\;[23]$$

where $Area_{R,0}$ represents the peak areas of HSYA at the beginning and $Area_{i,x}$ is the sum of peak areas of reaction sample at different time point. HSYA solutions at pH 9.16 were subjected to HPLC analysis (Figure S2) after incubation for various durations and the peak areas of HSYA and the products were determined. The decrease in HSYA content with time was accompanied by an increase in the content of its degradation products 1 (291 and 376 nm) and 2 (280 and 383 nm) (Figure 5). According to the mass balance formula, the sum of peak areas of HSYA and products remained over 80% within about 12 h, indicating mass balance.

### 2.6. The HSYA Degradation Products and Proposed Degradation Pathway

Soon after HSYA was diluted in the buffer solution of pH 9.16, a red shift in absorbance from 404 nm to 426 nm was observed, suggesting an increase in the electron cloud density of the conjugated system following rapid ionization under alkaline conditions. Subsequently, a gradual decrease in absorbance at 426 nm was observed, which was accompanied by a gradual increase in absorbance at 300 and 380 nm (Figure S3) from the degradation products.

The HSYA samples for the stability study were subjected to UPLC–ESI-MS/MS analysis under both negative and positive ion modes. HSYA was detected by the ion peaks of [M + H]^+^ *m*/*z* = 613 and [M + H]^−^ *m*/*z* = 611. A neutral loss of 18 due to loss of one water molecule on C-1′ hydroxyl and a neutral loss of 120 due to C-5′-glucoside cross elimination [24] were observed. The degradation products of HSYA were detected under alkaline conditions by the ion peaks of [M + H]^+^ *m*/*z* = 627 and [M + H]^−^ *m*/*z* = 625. Similarly, a neutral loss of 120 due to C-5′-glucoside cross elimination was observed. The UPLC–ESI-MS/MS data indicated that products 1 and 2 were isomers, which had the same molecular ions but different UV absorbance and UPLC retention time (Table 3).

**Scheme 1:**
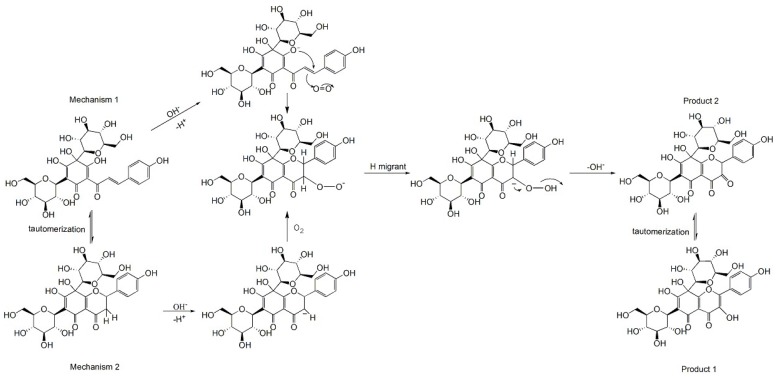
Pathway of HSYA degradation.

An HSYA degradation pathway was proposed on the basis of the MS ion fragmentation pattern and UV absorbance data obtained from the UPLC–ESI-MS/MS analysis. Under weak to moderate alkaline conditions (pH 7–9), the hydroxyl group at C-2′ is ionized firstly, followed by intra-molecular nucleophilic attack on the C_β_ of HSYA and [2 + 2] addition of O_2_ to the α, β unsaturated double bond. Finally, product 2 is formed after the hydrogen migration and the loss of [OH]^−^ [25,26]. The isomerization of product 2 results in the formation of product 1 as shown in Scheme 1.

The *k* in aqueous solution increased remarkably with pH under moderate alkaline conditions (pH 8–9), however, it decreased with pH under strong alkaline conditions (pH > 9.0). Chalcone, flavavone and the canban ion intermediate were all detected in the HSYA solutions. In agreement with previous findings [27], we found that the flavanone-chalcone isomerization equilibrium was regulated by alkalinity of the buffer. Both flavanone and chalcone were detected under moderate to strong alkaline conditions, while only flavanone was detected under very strong alkaline conditions (pH 13). Mechanism 2 in Scheme 1 demonstrates a proposed pathway of HSYA degradation under strong alkaline conditions.

### 2.7. Factors that Stabilize HSYA in XBJ Injection

We found that HSYA was liable to degradation in aqueous solution under alkaline conditions. However, it was much more stable in XBJ injection, which does not contain stabilizers. First, we examined the effects of the WSX fraction of XBJ injection on HSYA stability. HSYA and the other four active ingredients (PF, DSS, FA and Sen I) of XBJ were detected in EEX while dextrose was eluted in WSX. The HSYA stability in EEX and WSX was determined in pH 9.16 buffer solution to avoid the influence of different buffer salts. We also determined the effects of the four active ingredients, either alone or in combination on the *k* of HSYA in the buffer solution of pH 9 (Table 4).

**Figure 6:**
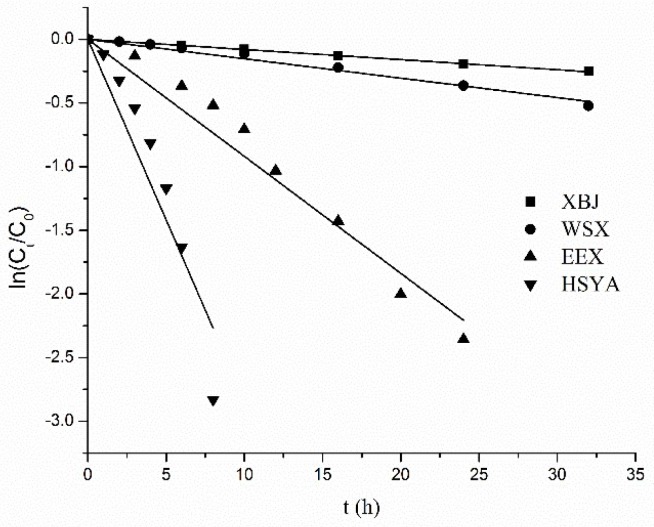
First-order plots for HSYA degradation under specified conditions.

As shown in Figure 6, HSYA reaction under all conditions tested followed the first-order reaction kinetics. As listed in Table 3, the *k* in EEX was relatively similar to that in XBJ injection; however, *k* in WSX was closer to that in aqueous solution. These results indicated that the water-soluble excipients such as dextrose had little influence on the stability of HSYA. Similarly, none of the four active ingredients in XBJ injection had significant stabilizing effects on HSYA.

**Figure 7:**
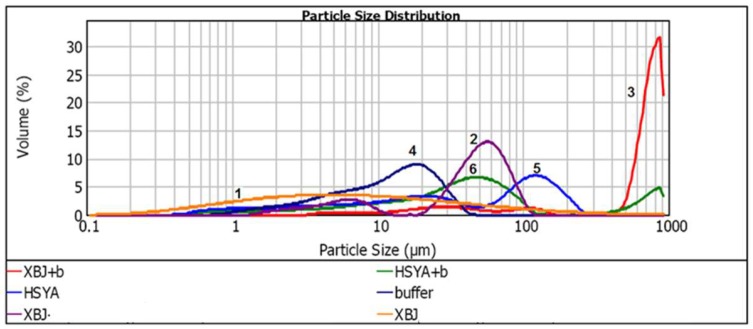
Particle size distribution of HSYA samples (1, XBJ injection; 2, XBJ in aqueous solution; 3, XBJ in buffer solution; 4, buffer; 5, HSYA in aqueous solution; 6, HSYA in buffer solution).

**Figure 8:**
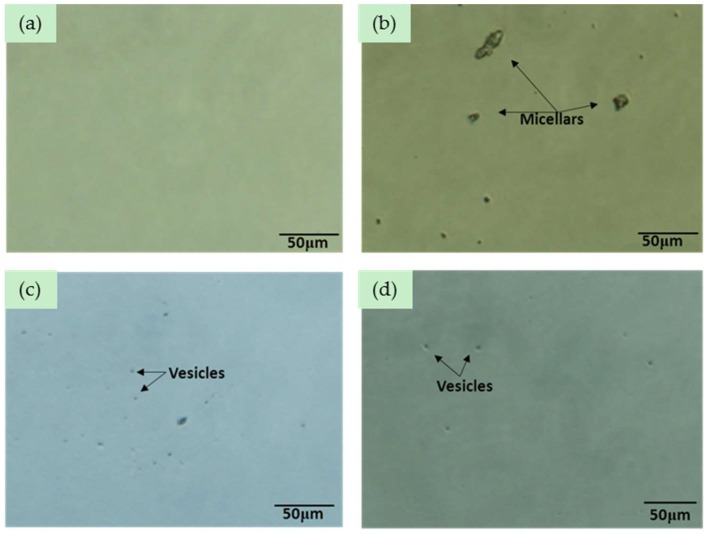
Inverted microscopic images of the original XBJ injection (**a**), buffer solution of XBJ injection (**b**), aqueous solution of XBJ injection (**c**) and HSYA in buffer solution (**d**).

Xuebijing (XBJ) is an agent made from the extracts of five Traditional Chinese Medicinal herbs, *Flosc Carthami, Radix Paeoniaec Rubra, Radix Salviae Miltiorrhizae, Radix Angelicae Sinensis, and Rhizoma Chuanxiong*. Saponins are in rich in Radix Angelicae Sinensis and Rhizoma Chuanxiong, which have the effects of analgesia and sedation, alleviate inflammation etc. Flavonoid glycosides, including HSYA, were abundant in Flosc Carthami. The XBJ injection rapidly turned turbid under alkaline conditions, suggesting formation of aggregates of amphipathic saponins, flavonoid glycosides self-assemble in aqueous solution and possibly other excipients in the injection [28]. Microscopic examination of HSYA samples revealed the presence of large particles (Figure 7). The vesicles were uniformly dispersed, likely resulting from the repulsive interactions between charged saponins and/or flavonoid glycosides. No aggregates were detected in the original XBJ injection, while particles with a size of 30–900 nm or greater in diameter were detected soon after the XBJ injection was diluted in alkaline buffer solution (Figure 8 and Table 5). The formation of aggregates may significantly affect intermolecular interactions in the sample [29]. Incorporation of HSYA into the micelles where it is surrounded by a less polar environment may protect it against hydrolytic degradation [30]. Moreover, water molecules may be immobilized near the micellar interface via hydrogen bonding to the hydroxyl groups, creating a barrier to negatively charged nucleophiles, such as OH^−^ ions. Based on these proposed mechanisms of HSYA stabilization, we speculate that conjugated glycosides with long side chains would provide good protection of highly hydrophilic compounds in XBJ injection.

## 3. Materials and Methods

### 3.1. Reagents

The HSYA standard was obtained from the National Institute for Food and Drug Control (Beijing, China; Batch No. 111637-201106). The XBJ injection was supplied by Tianjin Chase Sun Pharmaceutical Co., Ltd. (Tianjin, China; Batch No. 1208171). Acetonitrile (Sigma-Aldrich, St. Louis, MO, USA) was of high performance liquid chromatography (HPLC) grade. All other chemicals were of analytical grade.

### 3.2. HPLC Analysis

HPLC analysis of HSYA solutions was carried out on a Waters 2695 HPLC system coupled with a Waters 2998 Photodiode Array Detector (Milford, MA, USA). Samples (10 μL) were uploaded onto a Waters Symmetry^TM^ RP18 column (4.6 mm × 150 mm, 5 μm) thermo stated at 25 °C. The column was run using a gradient elution of 0.2% aqueous formic acid (A) and acetonitrile (B) as follows: 0–5 min, 5–15% B; 5–15 min, 15–20% B; 15–16 min, 20–40% B; 16–20 min, 40% B; flow rate, 1.0 mL·min^−1^. The column was re-equilibrated for 10 min prior to each injection. HSYA was detected by absorbance at 320 nm. This method allowed satisfactory chromatographic separation of HSYA with no significant interference noted (Figure S1).

The stock solution of HSYA was prepared by dissolving the HSYA standard in methanol at a concentration of 703 μg·mL^−1^. Calibration curve in the range of 2–102 μg·mL^−1^ was established by plotting the peak area versus the concentration of HSYA standard solution in methanol (*n* = 3). The precision of measurement was calculated as the relative standard deviation (RSD) at a single concentration of HSYA at 51 μg·mL^−1^. The accuracy of measurement was calculated as the percent extraction recovery (% accuracy = [detected concentration/nominal concentration] × 100%) [31] of samples spiked with an approximately equal volume of HSYA stock solution.

### 3.3. UPLC-Triple-Quattro/MS Method

A Waters LC–MS system equipped with a Waters ACQUITY UPLC^TM^ system and a Waters Quattro Premier XE MS spectrometer (Milford, MA, USA city) was used to analyze the HSYA degradation products. Chromatographic separation was carried out on a Waters Acquity UPLC BEH Shield C18 Column (2.1 mm × 100 mm, 1.7 μm) thermo stated at 40 °C. The column was run using a gradient elution of 0.1% formic acid–water (A) and acetonitrile (B) as follows: 0–2 min, 5–12% B; 2–8 min, 12–16% B; flow rate, 0.25 mL·min^−1^; auxiliary gas, 5 arbitrary units; sheath gas, 20 arbitrary units; spray voltage, 3.2 kV; capillary temperature, 120 °C. The mass spectrometer was programmed to perform full scans from 50 to 1000 *m*/*z*.

### 3.4. Sample Preparation

All glassware was sterilized by autoclaving for 20 min at 120 °C prior to use to avoid microorganism contamination. Phosphoric acid and trisodium phosphate were used to prepare the buffer solutions of different pHs (pH 1.44–12.73). The pH meter (Sartorius, Goettingen, Germany) was equipped with a combination electrode and calibrated with buffers of pH 4.01, 6.86 and 9.18. HSYA solutions at 45 μg·mL^−1^ were prepared by diluting the HSYA stock solution (703 μg·mL^−1^) or the XBJ injection (containing 448 μg·mL^−1^ of HSYA) in different buffers. These solutions were used for the HSYA stability study.

### 3.5. Measurement of HSYA Stability at Different pHs

HSYA solutions at 45 μg·mL^−1^ prepared as described in Section 2.4 were sealed in light-proof flasks and kept at 25 °C. Samples were periodically withdrawn and subjected to HPLC analysis to determine the remaining HSYA content. The observed rate constant (*k*) was calculated using the first-order rate equation. Linear regression analysis was performed using Microsoft Excel 2007 (Microsoft Corp., Redmond, WA, USA).

### 3.6. Measurement of HSYA Stability at Different Temperatures

The stability of HSYA at different temperatures was evaluated at neutral pH (6.8). HSYA solutions at 45 μg·mL^−1^ prepared as described in Section 2.4 were sealed in light-proof flasks and incubated in a water bath at 65 °C, 75 °C, 85 °C and 95 °C. Samples were periodically withdrawn, rapidly cooled to room temperature and subjected to HPLC analysis. The *k* was calculated using the first-order rate equation and the activation energy (*Ea*) of HSYA degradation was determined using the Arrhenius equation.

### 3.7. Characterization of Degradation Products

The HSYA solutions were subjected to UPLC–ESI/MS analysis to characterize the degradation products formed after incubation under different pHs and temperatures. In addition, UV-vis spectra (200–800 nm) of these samples were recorded using a UV spectrophotometer (TU-1900; Purkinje General, Beijing, China). The degradation products were identified on the basis of the MS and UV data. Furthermore, the mass balance of HSYA and its degradation products was determined and the mechanisms of HSYA degradation were proposed.

### 3.8. The Effects of XBJ Components on HSYA Stability

The XBJ injection contains no stabilizers other than dextrose, which is added to reduce the osmotic pressure. The XBJ injection was separated into EEX and WSX fractions by SPE chromatography. To investigate the effects of the WSX, which contains dextrose on HSYA stability, the XBJ injection (0.5 mL) was separated into two crude fractions on a needle type solid phase extraction column (SPE column, C18, 0.5 mL). The WSX fraction was eluted by 0.5 mL deionized water and the EEX was eluted by 1ml methanol. Next, the EEX fraction was concentrated under reduced pressure, dried with nitrogen and re-dissolved in 0.5 mL methanol. The HSYA concentration in EEX and WSX was determined and adjusted to 45 μg·mL^−1^ by adding the buffer of pH 9.16 (EEX) or HSYA stock solution (WSX). Samples were kept at 25 °C, withdrawn periodically and subjected to HPLC analysis. The *k* values of HSYA in EEX and WSX were determined and compared with those in aqueous solution and the original XBJ injection.

The effects of other bioactive ingredients (PF, DSS, FA, Sen I) in XBJ on HSYA stability were investigated in the buffer solution of pH 9. The *k* of HSYA was determined in the buffer solution of pH 9 in the presence of DSS, PF, FA, or Sen I, whose concentration was the same as that in the XBJ injection. The *k* was also determined in the presence of all four ingredients.

To investigate the effects of micelle formation on HSYA stability, the particle size of the aggregates formed in HSYA aqueous solutions under neutral and alkaline (pH 9.16) conditions and in XBJ injection was analyzed on an adopted Master sizer 2000 (Malvern Instruments Ltd, Malvern, UK) particle size analyzer.

The XBJ injection and HSYA solutions diluted in alkaline buffer solution (pH 9.16) or water as described above were transferred into a black 96-well assay plate with clear bottom. The samples were subjected to microscopic analysis with a Nikon ECLIPSE Ti-U Inverted Microscope (Nikon Instruments Inc., Melville, NY, USA) equipped with a DS-Fi1c high definition cooled color camera. Images were interpreted using Nikon NIS Elements Version 3.0 software (Melville, NY, USA).

## 4. Conclusions

The degradation of HYSA followed first-order kinetics in both aqueous solution and XBJ injection. The degradation rate showed an inverted V-shaped pH profile, with the greatest rate observed at appropriately pH 9. Under neutral pH (pH 7), ambient temperature (25 °C) and lucifugal conditions, HSYA was more stable in the XBJ injection than in the aqueous solution, especially under alkaline condition. The excellent stability of HSYA in XBJ injection partly due to the micelles formed in the injection to protect it against hydrolysis under alkaline conditions. Two HSYA degradation products were identified by UPLC–ESI/MS and the products were in mass balance. A reaction pathway was proposed accordingly in this paper. The study may provide clues for compatibility in TCM prescription and also provide useful information for preparation technology research of HSYA and assessment of clinical safety of XBJ.

## Figures and Tables

**Table 1:** Kinetic parameters of the hydrolysis of HSYA at 25 °C.

pH	HSYA in Aqueous Solution			HSYA in XBJ Injection		
	*k* (h^−1^)	r^2^	t_1/2_ (h)	*k* (h^−1^)	r^2^	t_1/2_ (h)
1.44	0.018	0.996	38.5	0.008	0.996	86.6
2.05	0.013	0.995	53.3	0.004	0.986	173.3
3.05	0.009	0.992	77.0	0.001	0.986	693.0
5.06	0.005	0.971	138.6	0.0008	0.992	866.3
6.13	0.001	0.985	693.0	0.001	0.980	693.0
7.10	0.003	0.987	231.0	0.005	0.975	693.0
8.15	0.051	0.984	13.6	0.005	0.996	138.6
8.55	0.061	0.989	11.4	0.007	0.984	138.6
9.16	0.283	0.910	2.4	0.008	0.990	99.0
9.50	0.247	0.992	2.8	0.013	0.991	77.0
10.15	0.215	0.991	3.2	0.011	0.990	53.3
10.50	0.114	0.985	6.1	0.011	0.991	63.0
10.95	0.104	0.983	6.7	0.008	0.981	63.0
11.56	0.081	0.99	8.6	0.008	0.989	86.6
12.73	0.023	0.985	30.1	0.008	0.996	86.6

**Table 2:** Kinetic parameters of HSYA degradation at different temperatures.

T/°C	HSYA in XBJ Injection			HSYA in Aqueous Solution		
	*k* (h^−1^)	R*^2^*	*Ea* (kJ∙mol^−1^)	*k* (h^−1^)	R*^2^*	*Ea* (kJ∙mol^−1^)
65	0.002	0.985	92.90	0.033	0.994	78.53
75	0.014	0.985		0.163	0.992	
85	0.021	0.971		0.203	0.983	
95	0.034	0.987		0.379	0.985	

**Table 3:** Mass, UV and UPLC retention time of HSYA and the products.

Compounds	t (min)	UV (λ, nm)	[M − H]^+^	[M − H]^−^	Fragmentations
HSYA	4.72	228,404	613	611	593, 491; [M − H_2_O]^−^, [M − C_4_H_8_O_4_]^−^
Product 1	3.08	290, 376	627	625	505; [M − C_4_H_8_O_4_]^−^
Product 2	2.49	280, 383	627	625	505; [M − C_4_H_8_O_4_]^−^

**Table 4:** Kinetic parameters of HSYA degradation under specified conditions.

	*k* (h^−1^)	r^2^	t_1/2_ (h)	t_0.9_ (h)
HSYA	0.283	0.910	2.45	0.37
WSX	0.091	0.963	7.62	1.15
EEX	0.015	0.981	46.2	7.03
XBJ	0.008	0.990	86.6	13.1
HSYA+mixture	0.136	0.942	5.10	0.78

**Table 5:** d(0.1), d(0.5) and d(0.9) values of particle size distribution of HSYA and XBJ in aqueous solution and alkaline buffer.

		Particle Size (μm)	
Samples	d(0.1)/μm	d(0.5)/μm	d(0.9)/μm
XBJ injection	0.851	6.116	58.531
XBJ + Water	5.201	47.210	78.927
XBJ + Buffer	35.759	729.872	894.720
Buffer	3.030	12.472	26.260
HSYA + Water	2.258	32.988	159.783
HSYA + Buffer	4.528	36.681	647.348

## Supplementary Materials

The supplementary materials are available online.
