# Supplementary material for: Superior Stability of Hydroxysafflor Yellow A in Xuebijing Injection and the Associated Mechanism
Source: Molecules. 2017 Dec 2;22(12):2129. doi: 10.3390/molecules22122129 (PMC6149961; doi:10.3390/molecules22122129)
Supplement: Supplementary file 1 [file molecules-22-02129-s001.pdf]

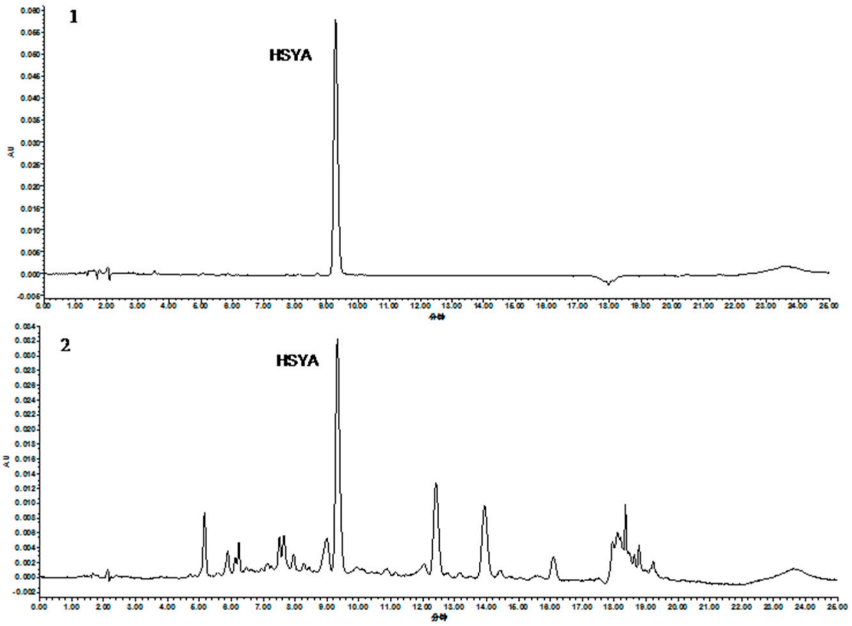

Figure S1 HPLC chromatogram of HSYA standard (1) and XBJ injection (2)

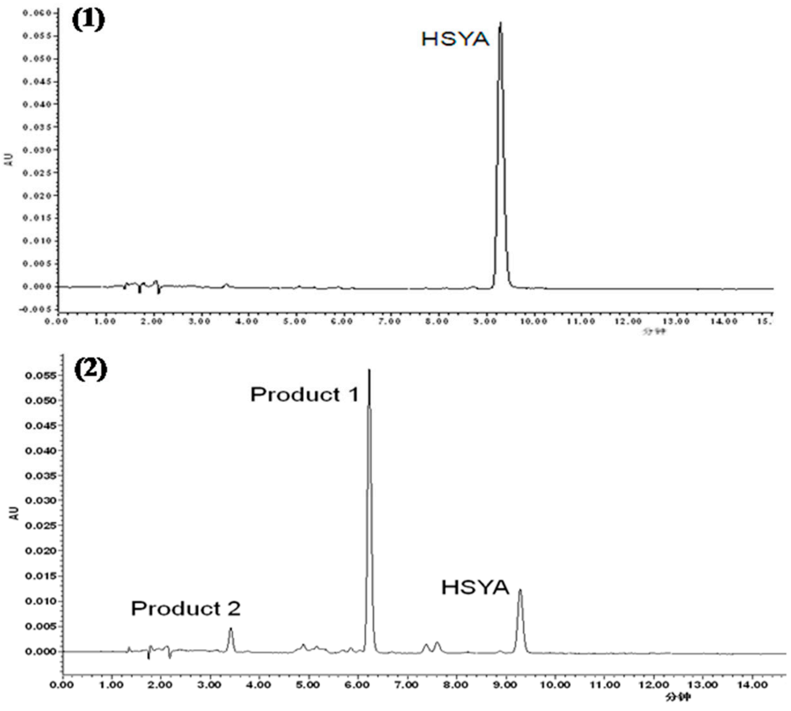

Figrue S2. HPLC chromatogram of HYSA standard (time = 0) (1) and HSYA in buffer solution of pH 9.16 after 7 h (2)

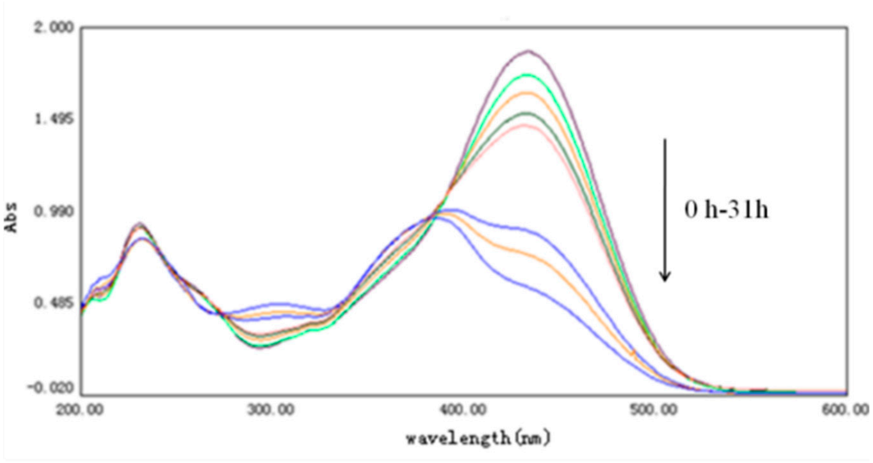

Figure S3. UV spectra of HSYA in alkaline solution in 0–3 h
